# Socio-Ecological Factors and Well-Being among Self-Employed in Europe during the COVID-19 Pandemic

**DOI:** 10.3390/ijerph19137840

**Published:** 2022-06-26

**Authors:** Josefine Hansson, Mikael Nordenmark, Åsa Tjulin, Bodil J. Landstad, Stig Vinberg

**Affiliations:** 1 Department of Health Sciences, Mid Sweden University, 831 25 Östersund, Sweden; mikael.nordenmark@miun.se (M.N.); asa.tjulin@miun.se (Å.T.); bodil.landstad@miun.se (B.J.L.); stig.vinberg@miun.se (S.V.); 2 Unit of Research, Education and Development, Östersund Hospital, 831 83 Östersund, Sweden

**Keywords:** self-employed, pandemic, socio-ecological model, small business, resilience, social support, useful work, clear rules

## Abstract

Background: The self-employed are at increased risk of negative well-being outcomes when facing adversity such as the COVID-19 pandemic. Studies that examine socio-ecological factors that may protect their well-being are warranted. Methods: Data were drawn from a cross-sectional survey of European self-employed people (*n* = 1665). The WHO-5 Well-being Index was used to examine the impact on well-being of factors at four socio-ecological levels. Independent sample *t*-tests, Pearson correlations and linear regression were applied to analyse differences between groups of self-employed and interactions between variables using SPSS. Results: Well-being and the socio-ecological factors of resilience, social support, useful work and finding the rules clear were positively correlated with well-being. For self-employed who reported that it was challenging to run their business during the pandemic, social support and finding rules clear were of significantly greater importance to their well-being. Conclusions: The findings highlight that the socio-ecological factors of resilience, social support, doing useful work and finding the rules clear affect well-being. The results also indicate that it is vital to consider factors at multiple socio-ecological levels to improve the well-being of the self-employed during adversity.

## 1. Introduction

The COVID-19 pandemic has affected the business activities of enterprises all around the world, causing pressures such as decreased customer demand, production stagnation, disruptions in supply chains and increased uncertainty [1,2]. Small businesses have been particularly affected, as they typically have fewer human, financial and technical resources compared to larger businesses [3]. In addition, gaps in specialised knowledge and limited managerial capacity (techniques, tools and procedures) may make it hard for them to handle the challenges brought about by the pandemic [4]. There is also evidence that small businesses have been less able to quickly adjust to changes in regulations and demands and have had difficulties in increasing their web presence, adding delivery services and handling uncertainty regarding liabilities [5]. However, even though the self-employed have faced significant challenges during the pandemic, studies have also reported resilience in the self-employed in terms of being agile, finding new business solutions and being optimistic about the future of their businesses [6,7,8].

The COVID-19 pandemic has not only had an influence on business operations and management but also on the well-being of the self-employed. For instance, a Swedish study of self-employed people during the pandemic found that increased stress and uncertainty about the future had a negative effect on their mental health and well-being [8]. Similarly, a large cross-sectional study reported increased uncertainty and health-related worries in the self-employed during the pandemic, which had a negative effect on their well-being [7]. Another study found that being in arrear was a significant problem for European self-employed people during the pandemic and that many were afraid to lose their homes due to being unable to pay the rent [9]. Research comparing the health and well-being of the self-employed with waged workers has yielded mixed results, with some studies reporting poorer well-being in the self-employed [10,11], whereas others state the opposite [12,13,14]. Having high levels of autonomy, flexibility, job satisfaction and many more advantages compared to waged workers [15,16] are commonly used as an explanation for better well-being in the self-employed. However, a recent critical review of the health of self-employed people states that these claims are outdated and that many self-employed earn low wages [16]. The health and well-being of the self-employed may be the most important intangible asset of a small business [17,18], and even the slightest health issue for a self-employed person can have detrimental consequences for the whole business [19]. As there is evidence that the well-being of self-employed people has been negatively affected by the pandemic, it is important to examine factors that may protect their well-being.

The socio-ecological model (SEM) is a holistic framework that conceptualises the linkages between individual behaviour and social and environmental determinants. The SEM recognises that an individual’s health is not only the product of biological factors but is influenced by various personal and environmental factors and the interplay between them [20]. According to the SEM, an individual’s behaviour is influenced by intrapersonal factors (e.g., knowledge, beliefs, attitudes and personality traits), interpersonal factors (e.g., social norms, peers and family,) organisational factors (e.g., environment and ethos), the community (cultural values and norms) and public policy (policies and laws at the local, regional or national level) [20]. This approach goes beyond simply individual influences on health and considers a variety of environmental factors that may also affect behaviour. In spite of the model being widely accepted [21], systematic reviews demonstrate that studies using the SEM rarely incorporate multi-level strategies, and few studies include the public policy level [22]. The focus instead tends to be directed toward the intrapersonal, interpersonal and organisational levels [21]. With regards to research on the self-employed during the pandemic, the vast amount of research appears to have focused on pathogenic effects at up to three of these levels [8,17,23]. However, there are also studies of the public policy level, such as the impact on the self-employed of governmental policies implemented during the pandemic [24] and international comparisons of support measures for businesses [25].

**Figure 1:**
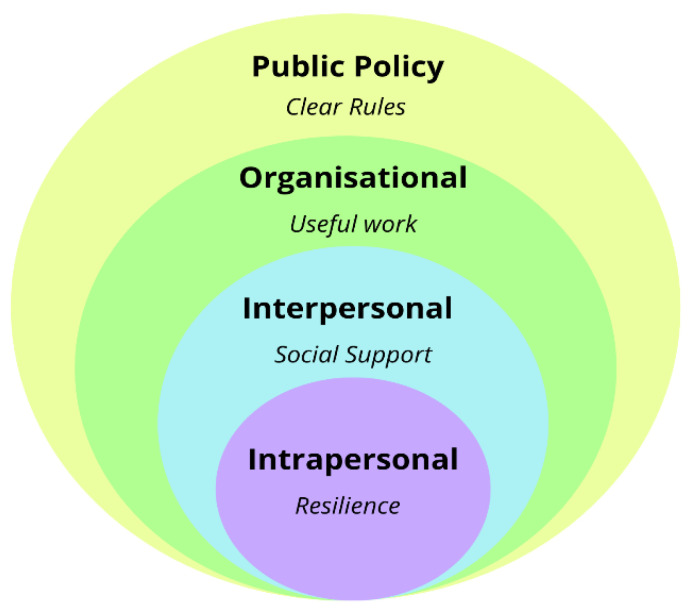
Four levels of socio-ecological factors that may influence the well-being of the self-employed during the pandemic. Adapted with permission from [37]. Copyright 1988 SAGE Publications.

Studies to date have provided evidence for the importance of factors at each level on the self-employed during the COVID-19 pandemic [6,7,8,26]. However, to our knowledge, no studies have simultaneously examined factors at all four socio-ecological levels. Additional studies are therefore needed to understand the range of socio-ecological factors contributing to the well-being of the self-employed in Europe during the pandemic. As illustrated in Figure 1, there is a possibility that the well-being of self-employed people may be affected by factors at four socio-ecological levels including intrapersonal (e.g., resilience), interpersonal (e.g., social support), organisational (e.g., doing useful work), and public policy (e.g., understanding rules of conduct).

At the intrapersonal level, resilience has been shown to protect the well-being of the self-employed during the pandemic [7]. There is a lack of consensus of the definition of resilience, and it is often equated with the concept of coping [27,28]. Resilience is generally described as the ability to bounce back from adversity and can be used to describe both an individual and a group’s response to challenging situations. In the present study, we use the definition “a protective factor that makes an individual more resilient (less vulnerable) to adverse events” as we strive to examine the complex interaction between risk factors and protective factors leading to positive health outcomes in the individual [29].

Social support, on the interpersonal level, has demonstrated to help the self-employed cope with stresses and protect their well-being during the pandemic [6,7,8]. Social support was in the present study defined as: “support accessible to an individual through social ties to other individuals, groups and the larger community” [30]. Doing meaningful and useful work, at the organisational level, is connected to well-being and physical health, and recently, more attention has been directed to the importance of these factors in the self-employed [26]. Doing useful and meaningful work is connected to work engagement, which has positive implications at a collective level in the form of commitment to the organisation [31,32]. Performing useful work is important for employees, as it gives meaning to the work [33]. During the pandemic, governments globally provided support measures for small businesses, and there is evidence that the eligibility for obtaining support, on the policy level, has been unclear for this group [34,35,36].

As mentioned, the consequences of the pandemic on the self-employed may vary depending on the degree to which they have been affected by the pandemic. Some businesses have adapted well to the challenges imposed by the pandemic, whereas others have suffered badly [6,7,23]. In the latter situation, higher scores on the various dimensions of the socio-ecological model may be even more beneficial to the level of well-being of the self-employed. In other words, it is likely that good socio-ecological conditions may provide even greater protection against ill health in precarious circumstances than if the situation were more stable [38,39,40]. The aim of the study was to investigate the well-being of self-employed people during the pandemic and whether their well-being was influenced by factors representing four socio-ecological levels: resilience, social support, doing useful work and clear rules. Furthermore, the study aimed to explore whether the four SEM factors would be more important for the self-employed who found it challenging to run their business during the pandemic compared to those who found it easy to run their business.

Research question 1 (RQ_1_): Are well-being and the SEM factors resilience, social support, doing useful work and finding the rules clear significantly better among the self-employed who find it easy to run their business compared to those who find it challenging to run their business?

Research question 2 (RQ_2_): Is the well-being of the self-employed during the pandemic significantly influenced by the four levels of SEM factors—the better the socio-ecological conditions the better the level of well-being?

Research question 3 (RQ_3_): Are the four levels of SEM factors more important to the self-employed who found it challenging to run their business during the pandemic compared to those who found it easy to run their business?

## 2. Materials and Methods

### 2.1. Study Design

A cross-sectional design was employed using data drawn from a survey entitled “Living, working and COVID-19” developed by the European Foundation for the Improvement of Living and Working Conditions (Eurofound) [41]. The majority of the questions were based on existing surveys, such as the European Quality of Life Survey (EQLS) [42] and the European Working Conditions Survey (EWCS) [33], while other questions were new or adapted from other sources, such as the EU Statistics on Income and Living Conditions (EU-SILC) [41]. The Eurofound data were made available to the authors of the present study with a confidentiality agreement on access to the dataset.

### 2.2. Data Collection

Data collection was performed between 22 June 2020 and 27 July 2020 via the SoSciSurvey platform [43]. A non-probability sampling method was applied, with survey participants being recruited using a snowballing technique distributed among Eurofound’s contacts and stakeholders. Complementary recruitment was conducted through advertisements on social media to target groups that were hard to reach. Due to the uncontrolled convenience sampling, the individual responses were re-weighted to be representative of the demographics of each respondent’s country [41]. Data were weighted by age crossed with gender, education level and urbanisation level. For each country, weighting targets based on population estimates from Eurostat by age and gender, education levels by age and gender from the Labour Force Survey, and self-defined urbanisation levels by age and gender as measured in the 4th EQLS were included. The anesrake R-package completed the weighting, and then the resulting weights were grossed up to population size. Lastly, the trimWeights function of the survey R-package trimmed extreme weights [41].

A total of 24,123 individuals from 27 EU countries participated in the study, of whom 1665 were self-employed. For this particular study, the sample consisted of self-employed with and without employees. In the questionnaire the question “Which of these categories best describes your situation?” was used, and two statements “self-employed with employees” and “self-employed without employees” were available to choose from. No option to specify the number of employees was given. However, approximately 93.0% of businesses in the European Union are micro-enterprises with less than 10 persons employed [44].

### 2.3. Measures 

#### 2.3.1. Outcome Variable

Well-being was measured using the WHO-5 Well-being Index, with 5 statements representing emotional and psychological stability over the past two weeks, including positive mood (good spirits, relaxation), vitality (being active and waking up fresh and rested), and general interest (being interested in things) [45]. The WHO-5 Well-being items were summarised in an additive index varying from 0 to 25, with higher scores indicating a higher level of well-being. This scale is commonly used across a wide range of study fields and has adequate validity both as a screening tool for depression and as an outcome measure in clinical trials [45]. The Cronbach alpha of the WHO-5 well-being index was 0.86, indicating a good level of reliability. Previous studies have also shown good Cronbach alpha values for the WHO-5 [46,47].

There is no clear definition of the concept of well-being, and the concept lacks clarity since similar terms are used interchangeably [48]. Furthermore, well-being means different things to different individuals, groups and cultures, hence it is difficult to reach a universally accepted definition of the concept [49]. In this study, we use the definition of well-being provided by the World Health Organisation (WHO), which considers well-being as another term for mental health [45]; “Mental health is a state of well-being in which an individual realizes his or her own abilities, can cope with the normal stresses of life, can work productively and is able to make a contribution to his or her community” [50].

#### 2.3.2. Exposure Variables

Socio-ecological factors were chosen based on aspects previously known to influence the well-being of the self-employed during the pandemic. These are psychological resilience, social support, doing useful work and understanding rules of conduct [6,7,8,26]. These were grouped at four ecological levels: intrapersonal, interpersonal, organisational and public policy.

##### Intrapersonal Level: Resilience

The participants’ psychological resilience was assessed using two statements adapted from the EQLS survey; “When things go wrong in my life, it generally takes me a long time to get back to normal”, and “I find it difficult to deal with important problems that come up in my life”. The scale ranged from 0 (Always) to 4 (Never), and a higher score indicated a higher level of psychological resilience. The resilience index was summarised into an additive index varying from 0 to 8, with higher scores indicating a higher level of well-being and had a Cronbach’s alpha of 0.76.

##### Interpersonal Level: Social Support

Participants reported their perceived level of social support from colleagues or peers (“Your colleagues or peers help and support you”) on a 5-point scale ranging from 0 (Never) to 4 (Always). The statement was adapted from the EQLS survey, and a higher score indicated a higher level of social support. 

##### Organisational Level: Useful Work

The statement “You have the feeling you are doing useful work” adapted from the EWCS was used to assess whether the participants experienced their work as meaningful. The scale ranged from 0 (Never) to 4 (Always), with a higher score indicating a higher level of meaningful work.

##### Policy Level: Rules for Obtaining Support Are Clear

To gain an insight into whether the support measures introduced during the pandemic were clear and transparent, the statement “The rules for obtaining support are clear and transparent” was used, and the scale ranged from 0 (Strongly disagree) to 4 (Strongly agree). A higher score indicated clearer and more transparent rules.

##### Difficulty to Run Business

The statement” I find it hard to bear the responsibility of running my business”, was used to distinguish whether the self-employed considered it challenging to run their business during the pandemic or not. The variable was named difficulty, and the scale ranged from 1 (Strongly agree) to 5 (Strongly disagree). A higher score indicated it easy to bear the responsibility of running the business. This variable was dichotomised in the following way: Strongly agree and agree = find it challenging (=0) and Neither agree nor disagree, Disagree and Strongly disagree = find it easy (=1).

##### Interaction Variables

Four interaction variables were calculated between the difficulty variable and the SEM variables. The variables were calculated by multiplying the difficulty variable with each of the four SEM variables. These variables were called Difficulty * Resilience, Difficulty * Social, Difficulty * Useful and Difficulty * Rules.

#### 2.3.3. Covariates

The covariates in the analyses included gender, age, education, sector of work, partner/spouse in household and being self-employed with or without employees.

Demographic characteristics included age (in years) and gender (male = 1, female = 0). Socio-economic factors comprised level of education (primary/secondary or tertiary), sector of work (agriculture, industry, transportation and construction = 1, service industry = 0), the presence of a partner/spouse in household (yes = 1, no = 0) and being self-employed with or without employees (with employees = 1, without employees = 0).

### 2.4. Statistical Analyses

Descriptive statistics were computed for background data using percentages and mean values. Independent sample *t*-tests assessed for differences between participants in the analytic sample. Linear correlations were examined for all variables. In order to determine the assumptions for the regression analysis, Cronbach’s alpha values were calculated to control the internal consistency of the multiple-item scales of well-being and resilience. A series of regression analyses, including interaction analyses, were performed to examine the relationships between the exposure variables and the outcome variable.

## 3. Results

### Study Sample

As shown in Table 1, the study sample consisted of 1665 self-employed people, of whom 47.9% were women. The mean age was 49.1 years (SD = 12.8) and 63.4% had a primary or secondary level of education. The majority, 80.8%, worked in the service industry, 71.5% had a partner/spouse and 70.9% were self-employed without employees. Of the group that found it more challenging to run their business, more were female (53.8%), more had a lower mean age (48.2), fewer had tertiary education (27.3%) and slightly fewer had a partner/spouse (69.6%) compared to those reporting it being easy to run their businesses.

The results from the independent sample *t*-tests examining RQ_1_ are shown in Table 2. The group that did not find it challenging to run their business during the pandemic had a significantly higher level of well-being (M = 14.32, *p* < 0.001) compared to the group that found it more challenging (M = 11.22, *p* < 0.001). Regarding three of the SEM factors, there were significant differences between the group that found it easy to run their business compared to the group that found it challenging. Resilience (M = 5.39, *p* < 0.001), social support (M = 2.39, *p* < 0.001), and finding the rules clear (M = 1.64, *p* < 0.001) were significantly higher in the group that found it easy compared to the group that found it challenging (resilience (M = 4.71, *p* < 0.001), social support (M = 1.93, *p* < 0.001), and finding the rules clear (M = 1.37, *p* < 0.001)). The mean value for doing useful work was higher in the group that found it easy to run their business but was not significant at the 0.05 level (*p* = 0.098).

Table 3 shows the correlations between well-being and socio-ecological factors. There is a statistically positive correlation between well-being and resilience (r = 0.440, *p* < 0.001), social support (r = 0.322, *p* < 0.001), useful work (r = 0.274, *p* < 0.001) and clear rules (r = 0.440, *p* < 0.001). Statistically positive correlations between the separate SEM variables were also found, with the exception of useful work and clear rules (r = −0.008).

Model 1 in Table 4 examines RQ_2_: The well-being of the self-employed would be significantly influenced by the SEM factors. The results indicate that the level of difficulty to run the business is significantly positively related to the well-being of the whole group (β = 1.57, *p* < 0.001). The SEM factors resilience (β = 1.16, *p* < 0.001), social support (β = 0.88, *p* < 0.001), doing useful work (β = 0.68, *p* < 0.001) and clear rules (β = 0.72, *p* < 0.001) are all positively correlated with well-being, with resilience having the strongest relation. These results mean that the higher the scores on the SEM factors, the better the well-being. When controlling for background factors, it can be observed that age, gender, education and having/not having employees do not correlate with well-being. However, having a spouse/partner has a positive correlation with well-being (β = 1.28, *p* < 0.001), whereas working in the service industry has a negative impact on well-being (β = −1.52, *p* < 0.001). The exposure and background variables explain 38% of the variance in well-being.

Models 2 to 4 in Table 4 examine RQ_3_: The four SEM factors will be more important to the self-employed who found it challenging to run their business during the pandemic compared to those who found it easy to run their business.

Model 2 indicates that for the group that found it easy to run their business, the SEM factors resilience (β = 1.21, *p* < 0.001), social support (β = 0.66, *p* < 0.001), useful work (β = 0.69, *p* < 0.001) and clear rules (β = 0.29, *p* < 0.05) are significantly positively related to well-being. In this group, the most important factors to well-being are resilience, useful work and social support. Having a spouse/partner (β = 0.66, *p* < 0.05) and age (β = 0.02, *p* < 0.05) are positively related to well-being while being self-employed with employees (β = −1.32, *p* < 0.001), working in the service industry (β = −1.04, *p* < 0.01), and being a self-employed female (β = −0.27, *p* < 0.01) have a negative correlation with well-being. The exposure and background variables explain 29% of the variance in well-being. In Model 3, it is evident that for the group that found it challenging to run their business, the SEM factors resilience (β = 1.22, *p* < 0.001), social support (β = 1.17, *p* < 0.001), useful work (β = 0.97, *p* < 0.001) and clear rules (β = 1.29, *p* < 0.001) are significantly positively related to well-being, and these variables together with the background variables explain 48% of the variance. The most important factor for this group is clarity in the rules for obtaining support, but all SEM factors are important to well-being. Having a spouse/partner has a positive effect on well-being (β = 1.49, *p* < 0.001), whereas working in the service industry has a negative correlation with well-being (β = −2.13, *p* < 0.001).

For both groups, resilience, social support, useful work and finding the rules clear have a positive influence on well-being. However, for the group that found it challenging to run their business during the pandemic, all these factors are of greater importance to their well-being. This is indicated, for instance, by the substantially greater R^2^ value in Model 3 than in Model 2.

However, it is difficult to tell from the analyses in Models 2 and 3 which factors are of significantly more importance for the group that found it challenging to run their business. Therefore, this will be further analysed in Model 4 by introducing the interaction variables. Analyses of interaction variables in Model 4 indicate that of the four SEM factors, social support (β = 0.74, *p* < 0.001) and clear rules (β = −0.98, *p* < 0.001) are of significantly greater importance for well-being among self-employed who found it challenging to run their business compared to those who found it easy to run their business.

## 4. Discussion

This study investigated the well-being of self-employed people during the pandemic, and whether their well-being was influenced by factors representing four socio-ecological levels: resilience, social support, doing useful work and clear rules. Further, the study explored whether the four SEM factors would be more important for the self-employed who found it challenging to run their business during the pandemic compared to those who did not find it more challenging to run their business. The results will be discussed in order of the main findings.

In support of research question 1, descriptive findings and independent sample *t*-tests confirmed that well-being and the SEM factors were significantly higher among self-employed who found it easy to run their business. The results are not surprising given the results from previous scholars indicating that self-employed who do well reported a good level of well-being during the pandemic [6] and that the aforementioned SEM factors were important to well-being [6,7,8,26]. However, what distinguishes this study from previous studies is the simultaneous application of socio-ecological factors at the intrapersonal, interpersonal, organisational and public policy levels. This approach was used in a previous study to predict mental health outcomes among healthcare workers during the COVID-19 pandemic [51], which highlights the importance of targeting multiple socio-ecological levels based on identified risk factors. Similarly, a study describing work-related health risks in small businesses [52] underscores the importance of including the social, economic and political contexts that influence individual decisions and opportunities in health promotion.

Resilience, social support, doing useful work and finding the rules clear all had a positive influence on the well-being of the self-employed, thus supporting research question 2—that the well-being of the self-employed was significantly influenced by the four SEM factors. Of these factors, the intrapersonal factor, resilience, had the largest positive impact on well-being for the whole group, indicating that resilience should be considered when studying factors that can support the self-employed during difficult times. Resilience has been shown to be important to well-being in previous research during the pandemic. For instance, a large global study found that psychological resilience helped the self-employed cope with stress and acted as protection for their well-being [7]. Similar results have also been found in the general population, with resilience reportedly acting as a protective influence against the adverse psychological effects of pandemic stressors [53,54,55]. Improving the resilience of the self-employed is important, as it enables individuals to thrive in stressful and adverse situations, preventing negative outcomes on well-being [56]. In order to build a person’s resilience, there must be adequate access to relevant resources as well as support in their surrounding environment [57]. Individual resources, such as self-efficacy, optimism and empathy are needed, but it is also important to take ecological resources into account. Ecological resources may include emotional and practical support at organisational and societal levels [58,59].

In this study, results from the two different groups of self-employed people support research question 3—that the SEM factors would be more important for the self-employed who found it challenging to run their business during the pandemic. For both groups, resilience, social support, doing useful work and finding the rules clear, which are factors at all four socio-ecological levels, correlated positively with well-being. However, for the group that found it challenging to run their business, all these factors were of greater importance to their well-being. The most important factor for this group was at the public policy level—that the rules for obtaining support were clear. It is possible that this was due to the fact that businesses that performed worse had a greater need to apply for financial aid than businesses that performed well [60]. Previous studies carried out during the pandemic reported that the self-employed found information regarding support measures unclear, and many were unsure of their eligibility for support [35,61]. Hence, policymakers must make the rules for obtaining support clearer in order to reduce ambiguity for the self-employed.

At an intrapersonal level, social support was positively correlated with well-being for both groups, but significantly more strongly correlated among self-employed people who found it hard to run their business. This is in congruence with other studies [6,7,8], which found social support to be an important element for the self-employed in coping with the COVID-19 pandemic. A plethora of research has demonstrated the protective effect of perceived social support on mental well-being in stressful situations [62,63], and it has been found that additional social support is needed in times of greater hardship [64]. This supports the result that social support was more important for the group that had a more difficult time running their business. Supporting the self-employed to make use of various social support resources in order to counteract the negative impact of adverse events on their well-being is important. Emotional support from family and friends [6,7] and discussing and exchanging information with peers have previously been shown to be important for coping with challenges both during the pandemic [6,7,8] and for everyday stresses [65]. The processes by which social support affects well-being are complex and span over multiple levels of the socio-ecological model [66]. For instance, social support may influence resilience [38] at an intrapersonal level or assist in making work meaningful [67] at an organisational level, which in turn may have a positive effect on an individual’s well-being.

Having the feeling of doing useful work was important for both groups of self-employed people. Previous research has stressed the importance of cultivating useful and meaningful work among managers during times of hardship and misfortune [40]. Work has a great impact on an individual’s physical and psychological health, and the feeling of doing useful work serves a valuable purpose for sustaining and enhancing well-being [68,69]. At an organisational level, it is important that the organisational culture has a shared pattern of values, meanings and assumptions about how things should be done every day in the organisation to create meaningfulness [70]. Self-employed people both with and without employees are in a position from which they can create opportunities for meaningful work, and creating a vision board linked to their perceived purpose and goals for the organisation may act as a driving force for fulfilment and self-actualisation [26].

In terms of future research, it may be valuable to investigate a greater number of factors at each level of the SEM, as the present study only included four factors when examining the impact on the well-being of the self-employed. Furthermore, the SEM may assist in the development of intervention studies seeking to include a more holistic approach in which the linkages between the behaviours of the self-employed and social and environmental determinants are taken into consideration.

### Limitations and Strengths

The study has some limitations that should be acknowledged. The individuals included in this study were recruited based on a non-probability sampling method and were not part of a randomly selected sample. However, due to the uncontrolled convenience sampling, the individual responses were re-weighted to represent the demographics of each respondent’s country [41]. Furthermore, a cross-sectional sample was used, making it hard to derive a causal relationship from the study [71].

One strength of the study is that the survey questions have also been used in other European studies [72] with validated questions and indices. The indices in our study show Cronbach’s alpha values of between 0.76 and 0.86, indicating satisfactory internal consistency. To our knowledge, this was the first study examining the effects of socio-ecological factors on the well-being of self-employed people.

## 5. Conclusions

This study found that well-being and the SEM factors of resilience, social support, doing useful work and finding the rules clear were significantly higher among the self-employed who found it easy to run their business during the pandemic. However, the SEM factors positively influenced the well-being of both groups, with resilience being particularly important to well-being. For the group that found it challenging to run their business, the SEM factors seem to be of greater importance for their well-being, which is indicated by the substantially greater R^2^ values for this group in the regression analyses. Analyses of the interaction variables showed that finding rules clear (on the policy level) and social support (on the interpersonal level) were of significantly more importance for the group finding it challenging. These results indicate that it is vital to support factors at multiple socio-ecological levels to improve the well-being of the self-employed and that it may be particularly important for those finding it more challenging to run their business during times of hardship.

## Figures and Tables

**Table 1:** Descriptive data for the study population (*N* = 1665).

Category	Managers, Total	Easy	Challenging
*n*	1665	1065	600
Gender, *n* (%)			
Male	867 (52.1)	590 (55.4)	277 (46.2)
Female	798 (47.9)	475 (44.6)	323 (53.8)
Age, *m* (SD)	49.1 (12.8)	49.6 (12.6)	48.2 (13.0)
Education, *n* (%)			
Primary or Secondary Education	1024 (63.4)	612 (58.4)	412 (72.7)
Tertiary Education	591 (36.6)	436 (41.6)	154 (27.3)
Sector of work			
Agriculture, industry, transportation, construction	315 (19.2)	199 (19.1)	116 (19.4)
Service industry	1326 (80.8)	844 (80.9)	482 (80.6)
Partner/spouse in household, *m* (%)			
Yes	1177 (71.5)	765 (72.6)	412 (69.6)
No	468 (28.5)	289 (27.4)	179 (30,4)
Self-employed with or without employees			
Without employees	1180 (70.9)	780 (73.2)	400 (66.7)
With employees	485 (29.1)	286 (26.8)	200 (33.3)

Note: Internal losses. Education is missing 50 values, sector of work is missing 24 values and partner/spouse is missing 20 values.

**Table 2:** Independent samples *t*-tests. Samples descriptive using *t*-tests (*N* = 1665).

	Easy		Challenging			
	M	SD	M	SD	T	Sig.
Well-being	14.32	4.72	11.22	5.45	12.10	0.001
Resilience	5.39	1.73	4.71	1.89	−7.32	0.001
Social support	2.39	1.18	1.93	1.23	−6.96	0.001
Useful work	3.01	0.87	2.93	1.00	−1.65	0.098
Clear rules	1.64	1.25	1.37	1.23	−4.06	0.001

Note: M = mean. SD = standard deviation. Easy = easy to run business. Challenging = more challenging to run business. T = t-value. Sig. = significance level.

**Table 3:** Correlations between main variables (Pearson) (*N* = 1665).

	1	2	3	4	5
Well-being	__				
Resilience	0.440 **	__			
Social support	0.322 **	0.140 **	__		
Useful work	0.274 **	0.192 **	0.222 **	__	
Clear rules	0.233 **	0.073 **	0.192 **	−0.008	__

** *p* < 0.01 level. Correlations = r.

**Table 4:** OLS Regressions for Well-being, Difficulty, SEM variables and Covariates (Unstandardised B-coefficients, Standard error in brackets).

Exposure Variable	Model 1 ^a^	Model 2 ^b^	Model 3 ^c^	Model 4 ^d^
Constant	1.60 (0.99)	3.95 (1.24) ***	−0.148 (1.62)	−0.34 (1.18)
Difficulty	1.57 (0.25) ***			
Resilience	1.16 (0.07) ***	1.21 (0.09) ***	1.22 (0.11) ***	0.93 (0.22) ***
Social support	0.88 (0.11) ***	0.66 (0.14) ***	1.17 (0.18) ***	2.05 (0.36) ***
Useful work	0.68 (0.13) ***	0.69 (0.19) ***	0.97 (0.19) ***	1.21 (0.41) **
Clear rules	0.76 (0.10) ***	0.29 (0.12) *	1.29 (0.15) ***	2.29 (0.33) ***
Difficulty * Resilience				0,14 (0.13)
Difficulty * Social				−0.74 (0.21) ***
Difficulty * Useful				−0.27 (0,26)
Difficulty * Rules				−0.98 (0.20) ***
Age	0.01 (0.01)	0.03 (0.01) *	−0.02 (0.02)	0.01 (0.01)
Gender	0.07 (0.25)	−0.27 (0.31) **	0.67 (0.39)	0.17 (0.24)
Spouse/partner	1.28 *** (0.27)	0.66 (0.34) *	1.49 (0.43) ***	1.12 (0.27) ***
Education	−0.17 (0.25)	−0.04 (0.30)	0.34 (0.45)	0.09 (0.25)
Sector	−1.52 *** (0.31)	−1.04 (0.39) **	−2.13 (0.49) ***	−1.51 (0.31) ***
Number SE	−0.45 (0.26)	−1.33 (0.33) ***	0.21 (0.43)	−0.67 (0.26) *
R^2^	0.38	0.29	0.48	0.41

*** *p* < 0.001 ** *p* < 0.01 * *p* < 0.05. ^a^ = regression of whole group, all variables. ^b^ = regression of the “easy” group, all variables. ^c^ = regression of the “challenging group”, all variables. ^d^ = regression with interaction variables.

## Data Availability

Restrictions apply to the availability of these data. Data were obtained from Eurofound [73], and are available from the authors with the permission of Eurofound.

## References

[1] Ma Z., Liu Y., Gao Y. (2021). Research on the impact of COVID-19 on Chinese small and medium-sized enterprises: Evidence from Beijing. PLoS ONE.

[2] Eurofound (2021). Business Not as Usual: How EU Companies Adapted to the COVID-19 Pandemic.

[3] Martin D., Romero I., Wegner D. (2019). Individual, Organizational, and Institutional Determinants of Formal and Informal Inter-Firm Cooperation in SMEs. J. Small Bus. Manag..

[4] Klein V.B., Todesco J.L. (2021). COVID-19 crisis and SMEs responses: The role of digital transformation. Knowl. Process. Manag..

[5] Fairlie R., Fossen F.M. (2022). The early impacts of the COVID-19 pandemic on business sales. Small Bus. Econ..

[6] Hansson J., Landstad B.J., Vinberg S., Hedlund M., Tjulin Å. (2022). Small business managers and COVID-19—The role of a sense of coherence and general resistance resources in coping with stressors. PLoS ONE.

[7] Stephan U., Zbierowski P., Pérez-Luño A., Klausen A., Alba Cabañas M., Barki E., Benzari A., Bernhard-Oettel C., Boekhorst J., Dash A. (2021). Entrepreneurship during the COVID-19 Pandemic: A Global Study of Entrepreneurs’ Challenges, Resilience, and Well-Being.

[8] Vinberg S., Danielsson P. (2021). Managers of micro-sized enterprises and COVID-19: Impact on business operations, work-life balance and well-being. Int. J. Circumpolar Health.

[9] Ahrendt D., Mascherini M., Leoncikas T., Sandor E. (2020). Living, Working and COVID-19: First Findings—April 2020.

[10] Patel P.C., Wolfe M.T., Williams T.A. (2019). Self-employment and allostatic load. J. Bus. Ventur..

[11] Lewin-Epstein N., Yuchtman-Yaar E. (1991). Health Risks of Self-Employment. Work. Occup..

[12] Stephan U., Roesler U. (2010). Health of entrepreneurs versus employees in a national representative sample. J. Occup. Organ. Psychol..

[13] Nikolova M. (2019). Switching to self-employment can be good for your health. J. Bus. Ventur..

[14] Binder M., Coad A. (2013). Life satisfaction and self-employment: A matching approach. Small Bus. Econ..

[15] Shir N., Nikolaev B.N., Wincent J. (2019). Entrepreneurship and well-being: The role of psychological autonomy, competence, and relatedness. J. Bus. Ventur..

[16] Khan T.H., MacEachen E., Hopwood P., Goyal J. (2021). Self-employment, work and health: A critical narrative review. Work.

[17] Torrès O., Benzari A., Fisch C., Mukerjee J., Swalhi A., Thurik R. (2022). Risk of burnout in French entrepreneurs during the COVID-19 crisis. Small Bus. Econ..

[18] Torrès O., Thurik R. (2019). Small business owners and health. Small Bus. Econ..

[19] Chao L.-W., Pauly M., Szrek H., Pereira N.S., Bundred F., Cross C., Gow J. (2007). Poor health kills small business: Illness and microenterprises in South Africa. Health Aff..

[20] Sallis J., Owen N., Fisher E. (2008). Ecological Models of Health Behavior. Health Behav. Health Educ..

[21] Kumar S., Quinn S.C., Kim K.H., Musa D., Hilyard K.M., Freimuth V.S. (2012). The Social Ecological Model as a Framework for Determinants of 2009 H1N1 Influenza Vaccine Uptake in the United States. Health Educ. Behav..

[22] Golden S.D., Earp J.A.L. (2012). Social Ecological Approaches to Individuals and Their Contexts. Health Educ. Behav..

[23] Vinberg S., Landstad B.J., Tjulin Å., Nordenmark M. (2021). Sickness Presenteeism among the Swedish Self-Employed During the COVID-19 Pandemic. Front. Psychol..

[24] Katare B., Marshall M.I., Valdivia C.B. (2021). Bend or break? Small business survival and strategies during the COVID-19 shock. Int. J. Disaster Risk Reduct..

[25] Tetlow G., Dalton G. (2020). Support for Business during the Coronavirus Crisis: An International Comparison.

[26] Geldenhuys D.J., Johnson S. (2021). Experience of meaningful work for self-employed individuals. SA J. Ind. Psychol..

[27] Van der Hallen R., Jongerling J., Godor B.P. (2020). Coping and resilience in adults: A cross-sectional network analysis. Anxiety Stress Coping.

[28] Pai N., Vella S.-L. (2018). Can one spring back from psychosis? The role of resilience in serious mental illness. Aust. N. Z. J. Psychiatry.

[29] Babić R., Babić M., Rastović P., Ćurlin M., Šimić J., Mandić K., Pavlović K. (2020). Resilience in Health and Illness. Psychiatr. Danub..

[30] Lin N., Simeone R.S., Ensel W.M., Kuo W. (1979). Social support, stressful life events, and illness: A model and an empirical test. J. Health Soc. Behav..

[31] Eurofound (2017). Sixth European Working Conditions Survey—Overview report (2017 Update).

[32] Rosso B., Dekas K., Wrzesniewski A. (2010). On the Meaning of Work: A Theoretical Integration and Review. Res. Organ. Behav..

[33] Eurofound (2019). European Working Conditions Surveys (EWCS): European Foundation for the Improvement of Living and Working Conditions. https://www.eurofound.europa.eu/surveys/european-working-conditions-surveys-ewcs.

[34] Belitski M., Guenther C., Kritikos A.S., Thurik R. (2022). Economic effects of the COVID-19 pandemic on entrepreneurship and small businesses. Small Bus. Econ..

[35] Blundell J., Machin S. (2020). Self-Employment in the COVID-19 Crisis: A CEP COVID-19 Analysis.

[36] Dörr J.O., Licht G., Murmann S. (2022). Small firms and the COVID-19 insolvency gap. Small Bus. Econ..

[37] McLeroy K.R., Bibeau D., Steckler A., Glanz K. (1988). An ecological perspective on health promotion programs. Health Educ. Q..

[38] Ozbay F., Johnson D.C., Dimoulas E., Morgan C.A., Charney D., Southwick S. (2007). Social support and resilience to stress: From neurobiology to clinical practice. Psychiatry.

[39] Wu G., Feder A., Cohen H., Kim J.J., Calderon S., Charney D.S., Mathé A.A. (2013). Understanding resilience. Front. Behav. Neurosci..

[40] Flotman A.-P. (2021). Work as Meaningful and Menacing Phenomenon for South African Middle Managers during the COVID-19 Pandemic: The Role of Self-Transcendence in Cultivating Meaning and Wellbeing. Front. Psychol..

[41] Eurofound (2020). Living, Working and COVID-19: Methodological Annex to Round 2.

[42] Eurofound (2016). European Quality of Life Surveys (EQLS): European Foundation for the Improvement of Living and Working Conditions. https://www.eurofound.europa.eu/surveys/european-quality-of-life-surveys.

[43] Leiner D.J. (2019). SoSci Survey [Computer Software].

[44] Eurostat (2019). Small and Medium-Sized Enterprises: An Overview: European Commission. https://ec.europa.eu/eurostat/en/web/products-eurostat-news/-/edn-20191125-1.

[45] Topp C.W., Østergaard S.D., Søndergaard S., Bech P. (2015). The WHO-5 Well-Being Index: A systematic review of the literature. Psychother. Psychosom..

[46] Wu S.-F.V. (2014). Rapid Screening of Psychological Well-Being of Patients with Chronic Illness: Reliability and Validity Test on WHO-5 and PHQ-9 Scales. Depress. Res. Treat..

[47] Omani-Samani R., Maroufizadeh S., Almasi-Hashiani A., Sepidarkish M., Amini P. (2019). The WHO-5 Well-Being Index: A Validation Study in People with Infertility. Iran J. Public Health.

[48] Kiefer R.A. (2008). An integrative review of the concept of well-being. Holist. Nurs. Pract..

[49] Faruk M.O., Alam F., Chowdhury K., Soron T.R. (2021). Validation of the Bangla WHO-5 Well-being Index. Glob. Ment. Health.

[50] WHO (2018). Mental Health: Strengthening Our Response. https://www.who.int/en/news-room/fact-sheets/detail/mental-health-strengthening-our-response.

[51] Hennein R., Mew E.J., Lowe S.R. (2021). Socio-ecological predictors of mental health outcomes among healthcare workers during the COVID-19 pandemic in the United States. PLoS ONE.

[52] Ingram M., Wolf A.M.A., López-Gálvez N.I., Griffin S.C., Beamer P.I. (2021). Proposing a social ecological approach to address disparities in occupational exposures and health for low-wage and minority workers employed in small businesses. J. Expo. Sci. Environ. Epidemiol..

[53] Chan A.C.Y., Piehler T.F., Ho G.W.K. (2021). Resilience and mental health during the COVID-19 pandemic: Findings from Minnesota and Hong Kong. J. Affect. Disord..

[54] Hou W.K., Tong H., Liang L., Li T.W., Liu H., Ben-Ezra M., Goodwin R., Lee T.M.-C. (2021). Probable anxiety and components of psychological resilience amid COVID-19: A population-based study. J. Affect. Disord..

[55] Riehm K.E., Brenneke S.G., Adams L.B., Gilan D., Lieb K., Kunzler A.M., Smail E.J., Holingue C., Stuart E.A., Kalb L.G. (2021). Association between psychological resilience and changes in mental distress during the COVID-19 pandemic. J. Affect. Disord..

[56] Rothstein M.G., Burke R.J. (2010). Self-Management and Leadership Development.

[57] Ungar M. (2011). The Social Ecology of Resilience: Addressing Contextual and Cultural Ambiguity of a Nascent Construct. Am. J. Orthopsychiatry.

[58] Foster K. (2020). Resilience in the face of adversity: A shared responsibility. Int. J. Ment. Health Nurs..

[59] McAllister M., Brien D.L. (2019). Empowerment Strategies for Nurses: Developing Resiliency in Practice.

[60] Block J.H., Kritikos A.S., Priem M., Stiel C. (2021). Emergency Aid for Self-Employed in the COVID-19 Pandemic: A Flash in the Pan?. SSRN Electron. J..

[61] Eib C., Berhard-Oettel C. (2020). Företagare under och efter COVID-19 [Entrepreneurs during and after COVID-19].

[62] Kawachi I. (2001). Social Ties and Mental Health. J. Urban Health Bull. NY Acad. Med..

[63] Eisman A.B., Stoddard S.A., Heinze J., Caldwell C.H., Zimmerman M.A. (2015). Depressive symptoms, social support, and violence exposure among urban youth: A longitudinal study of resilience. Dev. Psychol..

[64] Li F., Luo S., Mu W., Li Y., Ye L., Zheng X., Xu B., Ding Y., Ling P., Zhou M. (2021). Effects of sources of social support and resilience on the mental health of different age groups during the COVID-19 pandemic. BMC Psychiatry.

[65] Landstad B.J., Hedlund M., Vinberg S. (2017). How managers of small-scale enterprises can create a health promoting corporate culture. Int. J. Workplace Health Manag..

[66] Logie C., Lacombe-Duncan A., Lee-Foon N., Ryan S., Ramsay H. (2016). “It’s for us -newcomers, LGBTQ persons, and HIV-positive persons. You feel free to be”: A qualitative study exploring social support group participation among African and Caribbean lesbian, gay, bisexual and transgender newcomers and refugees in Toronto, Canada. BMC Int. Health Hum. Rights.

[67] Doenges T., Dik B., Banning J., Swaim R., Steger M. (2011). Calling and Meaningful Work among Student Military Veterans: Impact on Well-being and Experiences on Campus.

[68] Veltman A. (2015). Is meaningful work available to all people?. Philos. Soc. Crit..

[69] Johnson S., Robertson I., Cooper C. (2018). Well-Being. Productivity and Happiness at Work.

[70] Schein E.H. (2010). Organizational Culture and Leadership.

[71] Setia M.S. (2016). Methodology Series Module 3: Cross-sectional Studies. Indian J. Dermatol..

[72] Ahrendt D., Cabrita J., Clerici E., Hurley J., Leončikas T., Mascherini M., Riso S., Sándor E. (2020). Living, Working and COVID-19.

[73] Eurofound (2022). European Foundation for the Improvement of Living and Working Conditions. https://www.eurofound.europa.eu/.

